# Automatic Quantification of Computed Tomography Features in Acute Traumatic Brain Injury

**DOI:** 10.1089/neu.2018.6183

**Published:** 2019-05-22

**Authors:** Saurabh Jain, Thijs Vande Vyvere, Vasilis Terzopoulos, Diana Maria Sima, Eloy Roura, Andrew Maas, Guido Wilms, Jan Verheyden

**Affiliations:** ^1^ Research and Development, icometrix, Leuven, Belgium.; ^2^ Department of Radiology, Antwerp University Hospital and University of Antwerp, Antwerp, Belgium.; ^3^ Department of Neurosurgery, Antwerp University Hospital and University of Antwerp, Antwerp, Belgium.; ^4^ Department of Radiology, UZ Leuven, Leuven, Belgium.

**Keywords:** computed tomography, deep learning, quantification, traumatic brain injury

## Abstract

Traumatic brain injury is a complex and diverse medical condition with a high frequency of intracranial abnormalities. These can typically be visualized on a computed tomography (CT) scan, which provides important information for further patient management, such as the need for operative intervention. In order to quantify the extent of acute intracranial lesions and associated secondary injuries, such as midline shift and cisternal compression, visual assessment of CT images has limitations, including observer variability and lack of quantitative interpretation. Automated image analysis can quantify the extent of intracranial abnormalities and provide added value in routine clinical practice. In this article, we present icobrain, a fully automated method that reliably computes acute intracranial lesions volume based on deep learning, cistern volume, and midline shift on the noncontrast CT image of a patient. The accuracy of our method is evaluated on a subset of the multi-center data set from the CENTER-TBI (Collaborative European Neurotrauma Effectiveness Research in Traumatic Brain Injury) study for which expert annotations were used as a reference. Median volume differences between expert assessments and icobrain are 0.07 mL for acute intracranial lesions and −0.01 mL for cistern segmentation. Correlation between expert assessments and icobrain is 0.91 for volume of acute intracranial lesions and 0.94 for volume of the cisterns. For midline shift computations, median error is −0.22 mm, with a correlation of 0.93 with expert assessments.

## Introduction

Traumatic Brain injury (TBI) is a complex and often poorly understood disease process that is defined as an alteration in brain function, or other evidence of brain pathology, caused by an external force. Often referred to as “the silent epidemic,” it will surpass many diseases as the major cause of death and disability by 2020, according to the World Health Organization.^1^ In the acute phase after TBI, computed tomography (CT) imaging is commonly performed to detect the most important brain pathologies and quantify extent of injury. CT is widely available, fast, noninvasive, and remains the cornerstone for initial assessment and diagnosis of TBI in emergency settings.^2–4^ A broad spectrum of abnormalities/lesions can be encountered in TBI patients, depending on the type and amount of external forces that caused the initial insult. However, one of the most important purposes of imaging in the acute phase after injury is to identify the presence of large extra- or intracerebral space-occupying lesions that are in need of urgent neurosurgical evacuation (e.g., subdural hematomas, epidural hematomas, contusions, or intracerebral hematomas). The volume of these lesions and associated secondary features (a midline shift [MLS] greater than 5 mm, cisternal compression, etc.) are important guiding factors for surgical and medical management of raised intracranial pressure.^5,6^ In addition, some of these variables are also important for outcome prediction, which is why they are used in multiple prognostic CT scoring systems and are also commonly collected as important imaging variables in large-scale clinical TBI trials.^7–12^ Manual segmentation of these lesions is time-consuming and suffers from intra- and interobserver variability.^13–15^ A fully automated method could increase the reliability and consistency of volume estimations and quantification of associated factors (i.e., MLS and cisternal compression).

The aim of this article is to develop a fully automated method that could estimate acute intracranial lesion volume reliably and consistently quantify basal cistern volume and MLS. The main contributions of the article are: 1) application and extension of the U-Net–based convolutional neural network (CNN) for acute intracranial lesions segmentation; 2) accuracy validation of the proposed method on three subcohorts of the CENTER-TBI (Collaborative European Neurotrauma Effectiveness Research in Traumatic Brain Injury) data set, having multi-center data with different acquisition and imaging protocols.^7^

## Methods

### Data overview

In this article, data from the CENTER-TBI study (NCT02210221) is used, which recruited patients across a broad range of hospitals, including trauma centers, university hospitals, and community hospitals, over 5000 patients. The data are collected in three strata, differentiated by care path: 1) patients seen in the emergency room (ER) and discharged (ER stratum); 2) patients admitted to hospital, but not to the intensive care unit (ICU; admission stratum); (3) patients admitted to the ICU (ICU stratum). CT scan was performed according to standard clinical practice on either a GE (GE Healthcare, Little Chalfont, UK), Siemens (Siemens Healthcare, Erlangen, Germany), Philips (Philips Healthcare, Best, The Netherlands), or Toshiba (Toshiba Corporation, Tokyo, Japan) clinical scanner having a wide range of imaging (acquisition and reconstruction) parameters. Three distinctive subcohorts of the CENTER-TBI data set are considered for evaluating acute intracranial lesions segmentation: cistern segmentation and midline shift estimation such that every data set ensures a sufficient variability in terms of TBI severity and imaging characteristics of interest.

### Data set 1: Acute intracranial lesions delineation

The training data for acute intracranial lesions segmentation consist of 72 males and 33 females with 42 subdural hematomas, 43 epidural hematomas, and 66 intraparenchymal hemorrhages/contusions (multiple lesion types per patient were possible). The test data consist of 39 images and have similar distribution as the training, and the volumes range from 5.5 to 223 mL. Nine subjects were scanned on GE, 11 on Philips, 16 on Siemens, and three on Toshiba scanners. CT imaging parameters were as follows: computed tomography dose index (CTDIvol; in milliGrays) ranges from 0.03 to 85.66, peak kilovoltage (kVp) ranges from 80 to 140, slice thickness ranges from 0.41 to 5.00 mm, and pixel spacing ranges from 0.30 to 1.00 mm. Manual delineation of acute intracranial lesions is performed using 3D Slicer (version 4.8.1) (3D Slicer (online; accessed September 19, 2018; www.slicer.org)) by two neuroscientists (after mutual consensus), trained to interpret and segment TBI pathology. Each segmentation was supervised and validated by an expert neuroradiologist with over 25 years of experience.

### Data set 2: Cisterns delineation

Cistern data contain 70 cases with suprasellar, quadrigeminal, or prepontine/ambient cisternal compression indicated in the structured radiological reports. Multiple cisterns could be simultaneously compressed. Seven subjects were scanned on GE, 12 on Philips, 37 on Siemens, and 14 on Toshiba scanners. CT imaging parameters were as follows: CTDIvol (in milliGrays) ranges from 0.10 to 223.87, kVp ranges from 100 to 140, slice thickness ranges from 0.43 to 5.00 mm, and pixel spacing ranges from 0.29 to 1.00 mm. A (trained) neuroscientist manually segmented the cisterns using 3D Slicer under supervision of an expert neuroradiologist. Total volume of cisterns ranged from 0 to 19.76 mL.

### Data set 3: Midline shift measurement

MLS data contain 38 images for which the structured radiological reports indicated MLS status (<5 or >5 mm). Six subjects are scanned on the GE, 15 on Philips, 13 on Siemens, and 5 on Toshiba scanners. CT imaging parameters were as follows: CTDIvol (in milliGrays) ranges from 15.86 to 71.65, kVp ranges from 100 to 140, slice thickness ranges from 0.43 to 5.00 mm, and pixel spacing ranges from 0.35 to 1.00 mm. Two neuroradiographers (after mutual consensus) measured the MLS following the Common Data Elements,^16^ under supervision of an expert neuroradiologist. First, a line (line A) was drawn from the protuberantia occipitalis interna to the crista galli. At the level of the largest MLS, a line (line B) was drawn perpendicular to this line A, across the image, between the left and right internal table of the skull. A third line (line C) was drawn from the internal table of the skull to the septum pellucidum at the level of largest midline shift, in the opposite direction of the shift. This line was at the same level and parallel to line B. The MLS was then calculated with the following formula: ${ \vert \frac { B }  { 2 } - C \vert }$.^17^ Measurements were performed at all levels between the foramen of Monro and the roof of the lateral ventricles, where a possible MLS was visible. The measurement with the largest midline shift was taken as the final MLS. The experts' MLSs ranged from 0.20 to 19 mm.

### Method description

**FIG. 1.**
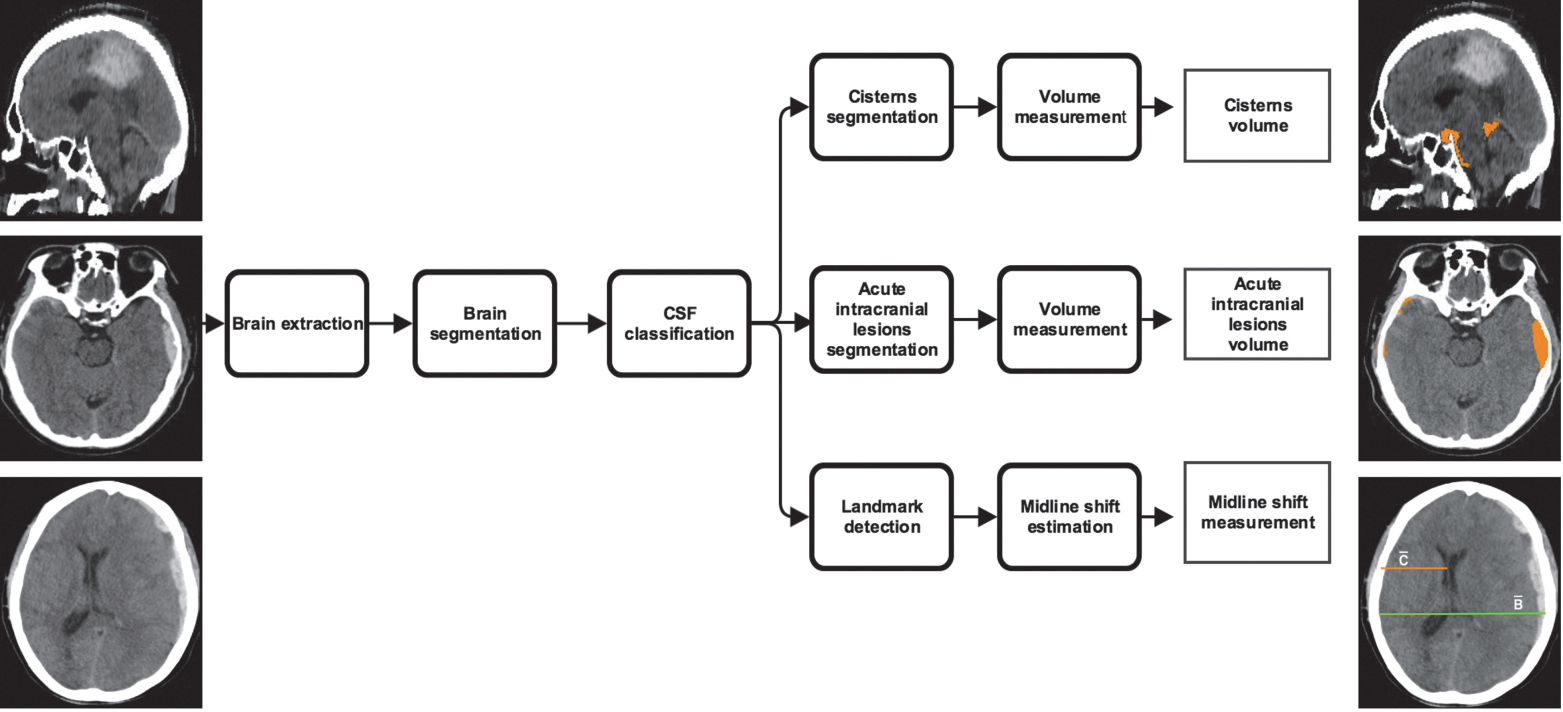
Schematic representation of icobrain method. CSF, cerebrospinal fluid. Color image is available online.

Figure 1 presents an overview of the proposed method, which has four steps. In the first step, the pipeline pre-processed the input CT image where the brain was extracted and segmented into gray matter, white matter, and cerebrospinal fluid (CSF). Brain extraction was performed using the 2D U-Net method described in Ronneberger and colleagues.^18^ The method contains five layers of contracting and expansive paths. In each layer, there are two convolutions of kernel size 3 × 3 and activated with a rectified linear unit (ReLu), which are normalized afterward with a zero mean and unit standard deviation. The number of filters used in the two convolutional blocks of the first layer were 64, and then in the successive layer, the number of filters was doubled. The network was optimized using Adadelta^19^ and was implemented in Python language (Python (online; accessed November, 28, 2018; https://www.python.org)) using Keras (Keras (online; accessed November, 28, 2018; https://keras.io)) with Tensorflow (Tensorflow (online; accessed November, 28, 2018; https://www.tensorflow.org)) backend. Brain segmentation was computed by registering the input image to the CT atlas^20^ in the Montreal Neurological Institute (MNI) coordinate space^21^ using NiftyReg,^22^ (NiftyReg (online; accessed November, 28, 2018; http://cmictig.cs.ucl.ac.uk/wiki/index.php/NiftyReg)) followed by segmenting the skull-stripped input CT image with the help of the available MNI CT before probability maps of gray matter, white matter, and CSF using a maximum likelihood expectation/maximization algorithm, NiftySeg. (NiftySeg (online; accessed November, 28, 2018; http://cmictig.cs.ucl.ac.uk/research/software/software-nifty/niftyseg)) The CSF segmentation was used to locate the lateral ventricles as well as the cisterns in the input CT image using binary anatomical priors of these structures available from MNI coordinate space.

**FIG. 2.**
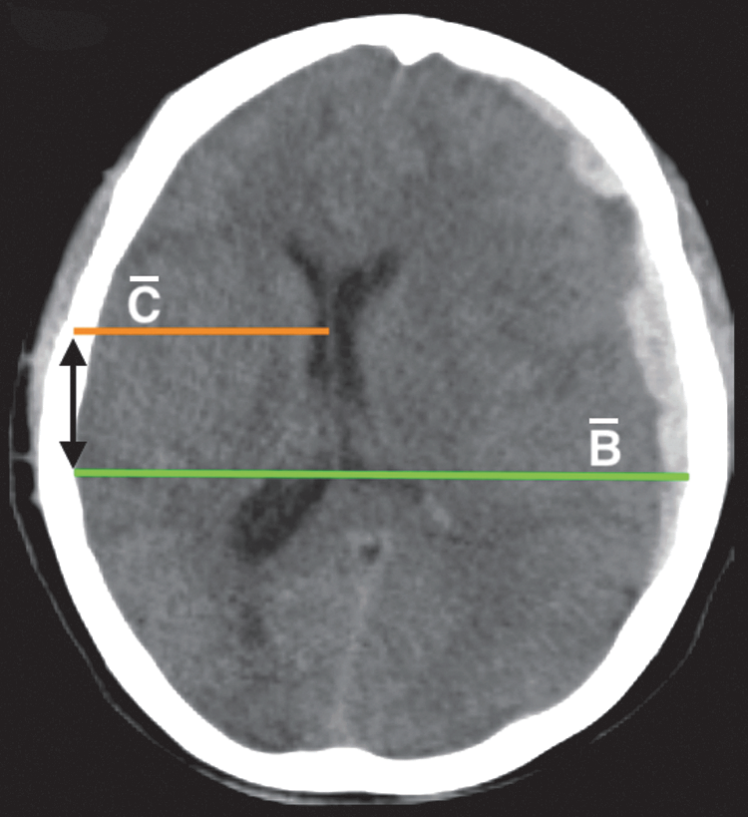
Estimation of the midline shift. ${ \bar B}$ is the max width in the axial plane and ${ \bar C}$ is the distance from the middle of the frontal part of the ventricles to the skull such that ${ \bar B}$ and ${ \bar C}$ have the same starting x coordinate value (black bidirectional arrow). The midline shift is then calculated using the expression: ${ \vert \frac { { \bar B } }  { 2 } - \bar C \vert }$. Color image is available online.

In the second step, cisterns were segmented by performing a set of morphological operations (constrained dilation, smoothing, and imposing nonoverlap at the cistern border) on the located cisterns from the pre-processing step to obtain the final segmentation. In the third step, acute intracranial lesions were segmented using a three-dimensional U-Net–based CNN architecture as described by Çiçek and colleagues.^23^ The CNN uses intensity-normalized (zero mean and unit standard deviation) extracted brain images from the pre-processing step for training. It extracts the relevant information from the input image and synthesizes the segmentation information at four resolutions (layers). In the extraction path, every layer has two 3 × 3 × 3 convolutions blocks, and each convolution is followed by a ReLu activation function. Then, a 2 × 2 × 2 max pooling operation decreases the input resolution by half in each dimension. The number of filters used in the two convolutional blocks of the first layer are 32 and 64, respectively, and then in the successive layer, the number of filters is doubled. During synthesis path, every layer has a 2 × 2 × 2 upsampling operation followed by two convolutions with ReLu, as described in the compression path. Shortcut connections are added from the same resolution of the compression path to provide high-resolution features before performing the convolution operation. In the final layer, a 1 × 1 × 1 convolution layer reduces the number of output channels to desired classes, followed by a softmax function to enforce sparse segmentation. The network is trained with an input voxel patch of the image of size 132 × 132 × 132 with desired output channels and a batch size of one. The output patch size is 44 × 44 × 44 and with a voxel size of 1 × 1 × 1 mm^3^; the approximate receptive field is approximately 88 × 88 × 88 mm^3^ for each voxel in the output segmentation. The network is trained with an Adam optimizer (learning rate = 10^–5^; decay factor = 0.0),^24^ with categorical cross entropy as a loss function,^#^ and is trained in two stages. In the first stage, the model is trained to differentiate intracranial lesions from the background using input and output image patches. 2) In the second stage, we computed the false lesions mask using the model segmentation from the first stage and the ground truth, and retrained the network with false lesions mask as an additional class. This forced the network to focus on the hard samples and learn to differentiate between acute intracranial lesions and false lesions. The false lesions mask includes venous sinus, free, and attached edges of the falx cerebri and tentorium cerebelli, etc., that contain blood, which have similar Hounsfield units as intracranial lesions. Because the data size is small, the images are augmented by flipping, translating, rotating, and adding Gaussian noise randomly. This would allow for more variability in the training set, and avoid possible overfitting. In the testing phase, the trained CNN assigns every voxel in an image a probability of being acute intracranial lesions, which is then thresholded to 0.7 (empirically chosen) to obtain the final segmentation. The network was implemented in Python language using Keras with Tensorflow backend. In the fourth step, the MLS was estimated by defining a range of potential slices between the foramen of Monro and the roof of the lateral ventricles in the MNI coordinate space. On each of these slices, the shift was calculated using the formula: ${ \vert \frac { { \bar B } }  { 2 } - \bar C \vert }$, where ${ \bar B}$ corresponds to the max width in the axial plane and ${ \bar C}$ is the distance from the middle of the frontal part of the ventricles to the skull such that ${ \bar B}$ and ${ \bar C}$ have the same starting x-coordinate value (Fig. 2). Maximum displacement is given as the MLS encountered for the patient.

### Performance metrics

The acute intracranial lesions and cisterns volume agreement between icobrain and expert reference segmentation was evaluated through the volume difference (in mL), absolute volume difference, and the intraclass correlation coefficient (ICC). Volume difference was computed as the difference between the value derived from the expert reference segmentation and the corresponding total volume reported by the icobrain method. A positive value indicates undersegmentation, and negative value indicates oversegmentation by the automated method. To measure the deviation from the expert reference segmentation (irrespective of under- or oversegmentation), absolute volume difference was computed, which was the absolute value of the volume difference. The ICC assessed the agreement of measurements made by multiple observers measuring the same quantity.^25^ In this article, ICC was used in the absolute agreement formulation.

The Dice similarity index was used to evaluate the overlap agreement between icobrain and expert reference segmentations for acute intracranial lesions and cistern segmentations. It was defined as the ratio between the number of voxels where both the icobrain and the expert reference segmentation agree (true positives; TP) and the mean number of voxels labeled as acute intracranial lesions/cisterns by the two methods.^26^ Segmentation quality was evaluated by sensitivity and precision. Sensitivity was defined as the ratio between TP and the total number of acute intracranial lesions/cisterns voxels in the expert reference segmentation (TP and false negatives [FN]). Precision was defined as the ratio between TP and the total number of acute intracranial lesions/cisterns voxels in the automatic segmentation (TP and false positives [FP]). Mathematically, Dice, sensitivity, and precision are defined as follows: $Dice = \; { \frac { 2 { \rm { TP } } }  { 2TP \; + \;FP \; + \;FN \; } } \;$, $Sensitivity = \; { \frac { { \rm { TP } } }  { TP \; + \;FN } }$, and $Precision = \; { \frac { { \rm { TP } } }  { TP \; + \;FP } }$. An additional classification accuracy measure is reported, defined as the ratio of number of cases where both reference and automatic measurements agree (i.e., their respective largest lesion volume is either >25 or <25 mL) and total number of subjects.

For the MLS, the difference in shift (in mm) was computed between the expert and icobrain method measurements. We also report the absolute shift measurement, which is the absolute value of the difference in shift. Finally, the classification accuracy measure is also reported. In this case, the agreement was defined where both reference and automatic measurements either measured the shift >5 or <5 mm.

## Results

### Quantitative results

**FIG. 3.**
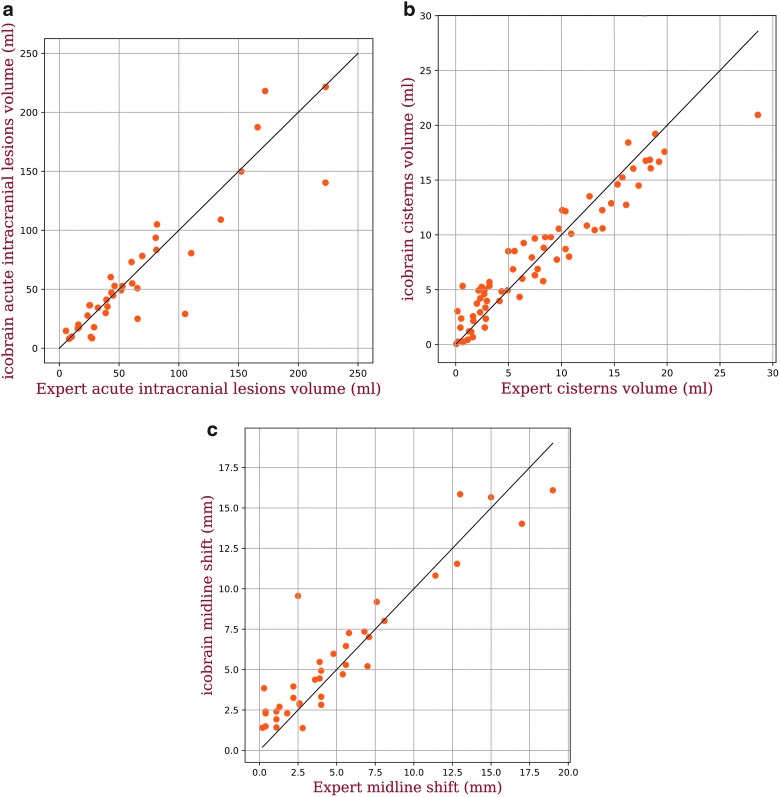
Scatter plots of expert reference values versus icobrain computed values for (**A**) total acute intracranial lesions volume, (**B**) total cisterns volume, and (**C**) midline shift. Color image is available online.

Table 1 presents the quantitative performance of the method for acute intracranial lesions, cisterns, and midline shift. For acute intracranial lesions, the median volume difference between icobrain and the expert reference segmentations was 0.07 mL, whereas the median value of absolute volume difference was 8.83 mL. icobrain acute intracranial lesions volumes were well correlated to the expert reference volumes with an ICC of 0.91. Median overlap with the reference expert segmentation was 0.73 with a median precision and sensitivity of 0.75 and 0.75, respectively. Classification accuracy based on the 25-mL cutoff was 0.92. Similarly, for cistern segmentation, median volume difference was −0.01 mL, whereas the median value of absolute volume difference was 1.48 mL. Very good correlation (ICC of 0.94) was observed. Median overlap with the reference expert segmentation was 0.70, whereas precision and sensitivity were 0.72 and 0.69, respectively, for cistern volume >5 mL (*n* = 39). For the MLS, the median difference in the shift was −0.22 mm, whereas the median value of absolute difference in the shift was 0.86 mm. Again, a very good correlation was obtained, with ICC of 0.93. Classification accuracy based on the 5-mm cutoff was 0.89. Figure 3 visualizes the volumetric correlations between the expert reference and icobrain measurements.

**Table 1.** Performance Measures between Icobrain and Expert Reference Values for Acute Intracranial Lesions and Cistern Volumes and for Midline Shift

	*Acute intracranial lesions*	*Cisterns*
Volume difference (mL)	0.07 (−7.73, −7.47)	–0.01 (−1.66, −1.21)
Absolute volume difference (mL)	8.83 (2.02–17.05)	1.48 (0.70–2.26)
Classification accuracy	0.92	—
ICC	0.91	0.94
Dice	0.73 (0.55–0.81)	0.70^*^ (0.66–0.73)
Precision	0.75 (0.63–0.87)	0.72^*^ (0.67–0.79)
Sensitivity	0.75 (0.61–0.84)	0.69^*^ (0.64–0.74)

	*Midline shift*	
Shift difference (mm)	–0.22 (−0.88, −0.76)	
Absolute shift difference (mm)	0.86 (0.37–1.48)	
Classification accuracy	0.89	
ICC	0.93	

^*^ For cisterns, Dice, sensitivity, and precision are reported for volume >5 mL.

Except classification accuracy and ICC, all other measures are presented in median (25^th^-75^th^) percentiles.

ICC, intraclass correlation coefficient.

### Qualitative results

**FIG. 4.**
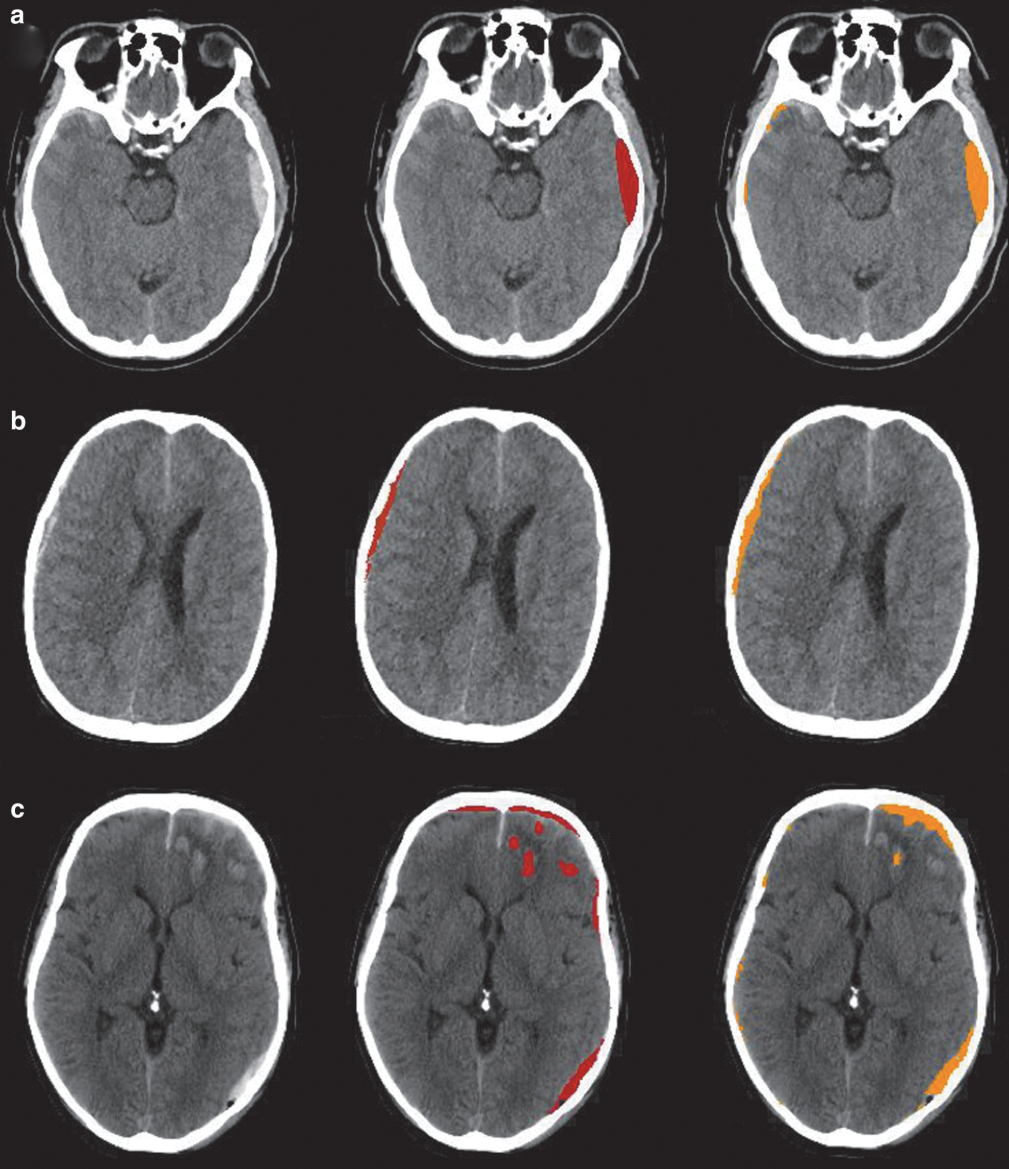
Qualitative results for acute intracranial lesions segmentation on 3 representative subjects from data set 1. The first column shows original computed tomography images with superimposed acute intracranial lesions segmentations from the expert in the second column and icobrain in the third column. (**A**) Acute intracranial lesions segmentation on a representative subject with Dice of 0.81, followed by (**B**) Dice of 0.73 and (**C**) Dice of 0.54. Color image is available online.

**FIG. 5.**
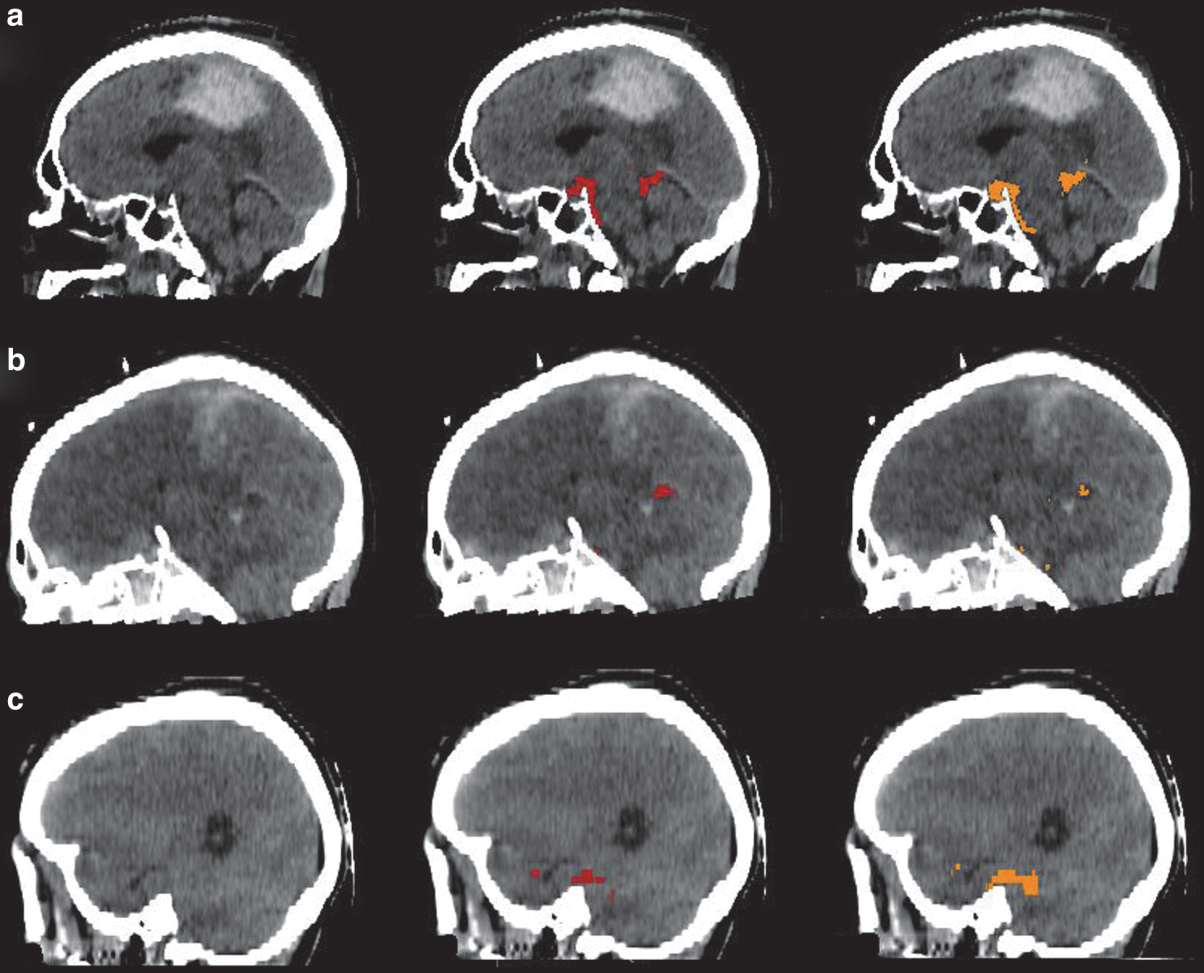
Qualitative results for cisterns segmentation on 3 representative subjects from data set 2. The first column shows original computed tomography images with superimposed cisterns segmentations from the expert in the second column and icobrain in the third column. (**A**) Cisterns segmentation on a representative subject with Dice of 0.73, followed by (**B**) Dice of 0.70 and (**C**) Dice of 0.66. Color image is available online.

**FIG. 6.**
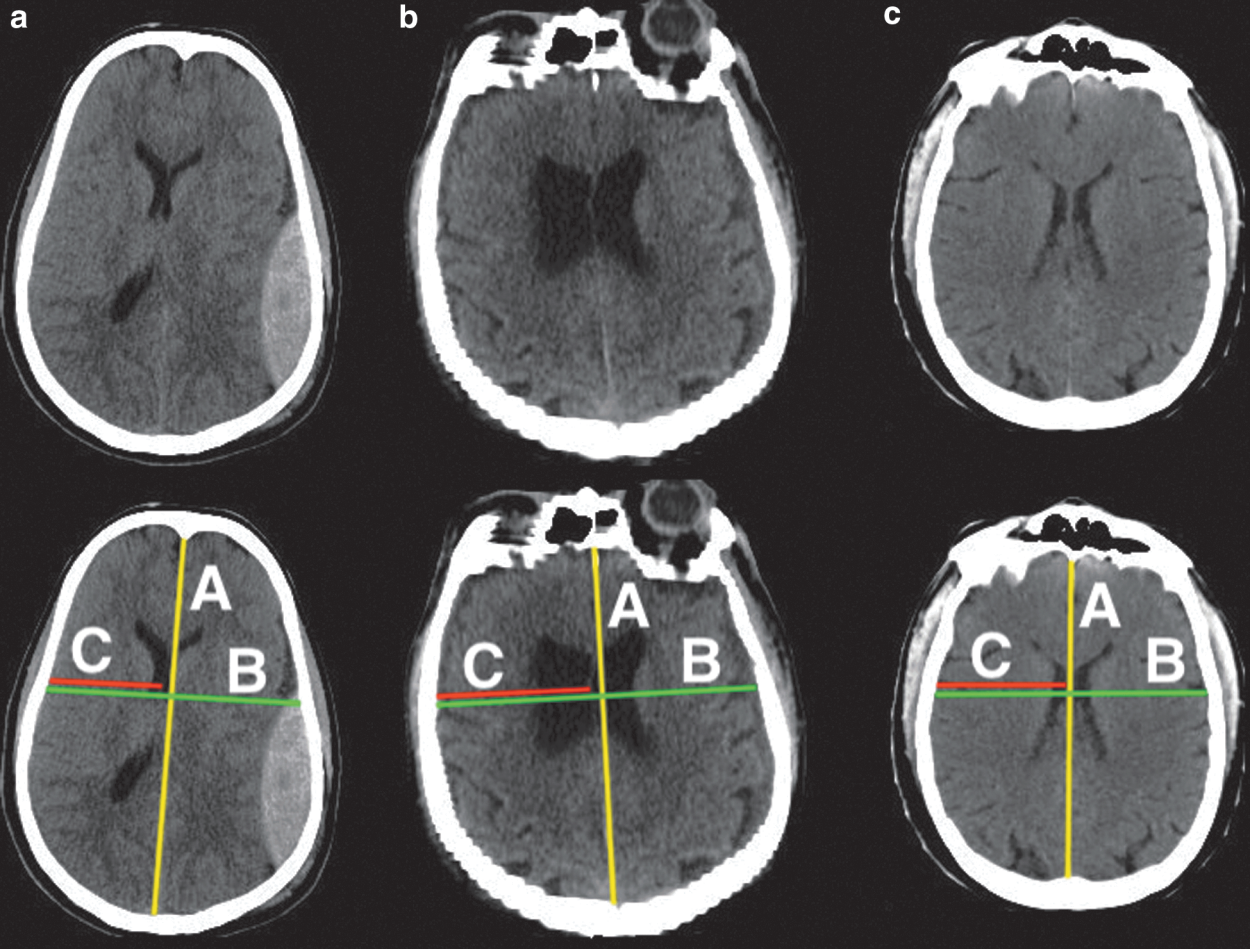
Qualitative results for midline shift estimation on 3 representative subjects from data set 3. First row presents original computed tomography images and second row shows how the midline is calculated by the expert. The estimated shifts (in mm) by the expert and icobrain for (**A**) is (5.60, 5.95), followed by (1.10, 1.91) for (**B**) and (0.40, 1.91) for (**C**). Color image is available online.

Figures 4 and 5 show several TBI cases from data sets 1 and 2, respectively, representative for the expected performance of the icobrain segmentations. The original CT images are shown next to annotated versions with the lesion segmentation from the expert reference segmentation (red) and icobrain (orange). The first row corresponds to the 75^th^ percentile of Dice, followed by 50^th^ and 25^th^ percentiles (Table 1) in the second and third rows, respectively. In Figure 4, epidural and subdural hematomas are well segmented with few false positives near the brain boundary. Isodense contusions (e.g., Fig. 4C) were difficult to segment accurately, especially if they were near the brain boundary. In Figure 5, cistern segmentations are similarly illustrated. The segmentations in Figure 5A show that the suprasellar and quadrigemnial cisterns match closely between the human expert and the automatic method, whereas the prepontine cistern differs in terms of the inferior stopping point, owing to the lack of a clear anatomical boundary. Figure 6 (second row) shows how the midline is measured by the expert and the corresponding shift obtained with the icobrain method on 3 subjects. The estimated shifts by the experts are 5.60 mm for Figure 6A, followed by 1.10 mm on Figure 6B and 0.40 mm for Figure 6C. The corresponding obtained values from the icobrain method are 5.95 mm for Figure 6A, followed by 1.91 mm for Figure 6B and 1.91 mm for Figure 6C.

## Discussion and Conclusion

In this article, icobrain is presented, a fully automated method that reliably computes acute intracranial lesions and cistern volumes along with MLS on a noncontrast CT image of a TBI patient. The method has been validated using a multi-center data set from the CENTER-TBI study.

Accurate volume estimation of acute intracranial lesions and cisterns could be of great added value in routine clinical practice. Unfortunately, their manual delineation is time-consuming and thus not often used in clinical practice. Semiquantitative methods, such as the ellipsoid method^27^ and the Cavalieri direct estimator,^28^ have been proposed to estimate volumes, but are still time-consuming and often lack precision, especially in lesions with irregular shapes. In addition, these methods are still observer dependent and are therefore subject to substantial inter- and intrarater variability.^29^ The same holds true for the interpretation of prognostically important features that are associated with large masses, like MLS and cisternal compression. For example, the state of the basal cisterns is currently still always qualitatively measured in clinical routine. Multiple semiquantitative methods could be used, which can be an additional source of variability.^30^

Automatic methods have the obvious advantage of being fast and consistent compared to manual and semiautomated methods. It is interesting to note that automatic MLS measurement has received considerable interest in the literature. Automatic methods that measure MLS either use symmetry of the brain or some specific anatomical landmarks, such as falx cerebri, frontal horns of the lateral ventricles, and the third ventricle.^31–35^ In general, all these methods perform well except in extreme cases, such as very large intracerebral hemorrhage (brain symmetry is almost always compromised).

For acute intracranial lesions segmentation, the techniques are based on intensity threshold, region growing, fuzzy clustering, active contours, or atlas registration.^36–40^ The data sets used in most of these methods are relatively small, with little information about variability in brain injury severity, which makes it difficult to compare them against our method. Small sample size in the data sets used in the literature could be attributed to the fact that acquiring and delineating lesions manually on large data sets is very challenging and time-consuming. Discriminating normal blood containing structures (e.g., venous sinuses) from intracranial lesions poses challenges for any automated method, given that they are in a close proximity and share the same range of image intensities in a noisy CT scan.

Comparing the performance of our method with the state-of-the-art methods is difficult not only because different data sets were used, but also because most of them focus on classification tasks (e.g., detecting MLS more than 5 mm), including classifying severity level of cistern effacement and detecting presence or absence of certain type of intracranial hemorrhages. This is indeed an important aspect; however, an accurate agreement of these parameters with the experts gives more insights in the method's performance and thus builds trust for any computer-based method.

For MLS, Chilamkurthy and colleagues^13^ evaluated their method for MLS detection >5 mm on a large data set and obtained an average sensitivity of 0.89 at a high specificity operating point. Wang and colleagues^41^ reported the accuracy of 0.90 on 43 subjects for MLS >5 mm. Our results are very similar, because we obtained a classification accuracy of 0.89 at the typical threshold of 5 mm. Yuh and colleagues^34^ assessed the MLS using symmetry of CSF in lateral ventricles with respect to the symmetry of the skull and observed the detection classification accuracy (for >5 mm shift) of 0.98 on 250 subjects obtained from multi-center data (vendor/acquisition parameters analysis was not performed). However, this study did not compare against expert measurements.

For intracranial lesion segmentation, Chilamkurthy and colleagues^13^ detected five different types of intracranial lesions and obtained an average sensitivity of >0.90 for each type. Similarly, Yuh and colleagues^34^ detected four different types of intracranial lesions and obtained good results, except for some difficulty in separating traumatic subarachnoid hemorrhage (tSAH) and contusions from the normal brain structures. Similar to MLS, the researchers classified different lesions, but unfortunately, no analysis (such as overlap with manual segmentation) was performed on their segmentation. Detecting different types of intracranial lesions is certainly important, and we aim to address this issue in our future work; nevertheless, we obtained a decent classification accuracy of 0.92 using a cutoff of 25 mL, which is a typical threshold for TBI lesions indicative of mass effect. Roy and colleagues^39^ performed segmentation analysis of intraparenchymal hemorrhages and reported a median overlap of 0.86 on 25 subjects, and we obtained a median overlap of 0.73 on 39 subjects. Although the results seem better, the lesion types were only a subset of intracranial lesions that we considered, and no information on distribution among subjects was given. Bhadauria and colleagues^36^ evaluated their method on 20 subjects and reported an 0.89 overlap with respect to expert ground truth. They considered 5 epidural, 4 subdural, and 11 intracerebral hemorrhage subjects, which is a much smaller data set than in our work (43 epidural, 42 subdural, and 66 intracerebral hemorrhages or contusions subjects).

**FIG. 7.**
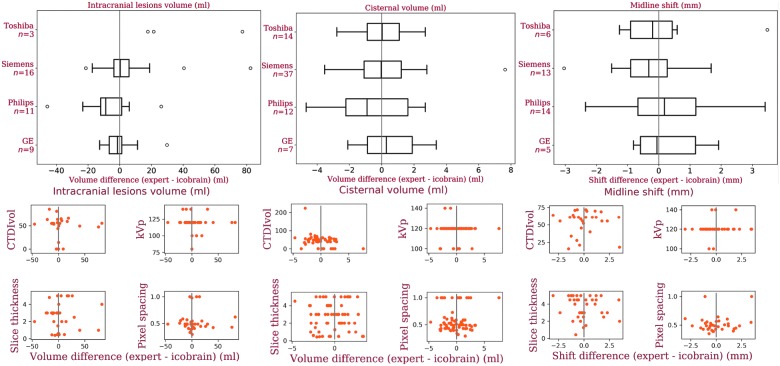
Analysis of icobrain's performance on four different manufactures and imaging parameters values. First column presents box plots and scatter plots reflecting the measurement error as a function of these hyperparameters for data set 1 followed by data set 2 and data set 3 in columnss two and three, respectively. CTDIvol = computed tomography dose index (in milliGrays); kVp = peak kilovoltage (in kV), slice thickness and pixel spacing are reported in mm. Color image is available online at www.liebertpub.com/neu

An important contribution of this work is the validation of our method on a multi-center data set. We investigated the performance of our method for different manufacturers and acquisition parameters (see Fig. 7). In summary, it seems that the performance of icobrain is not manufacturer dependent; however, the low sample size per manufacturer makes it difficult to generalize this conclusion. For imaging parameters, no trend was observed with the change in any parameter settings, which implies that the performance of icobrain is independent of imaging parameters values. Comparing with the literature, the median acute intracranial lesions volume difference between the expert reference segmentations and icobrain is 0.07 mL, and Jacobs and colleagues^14^ mentioned the median volume difference of −4.0 mL between the two experts on their data set. A low volume difference and slightly high absolute volume difference for acute intracranial lesions volume suggests that icobrain overestimates lower volumes and underestimates large volumes (see Fig. 3A) for acute intracranial lesions. Based on neurosurgical guidelines in Bullock and colleagues,^5^ the accuracy in computing the acute intracranial lesion volume between 25 and 50 mL is important, and our absolute volume difference was <25 mL. In 3 of 39 cases with the largest lesion volume >25 mL, we incorrectly measured the largest acute intracranial lesion volume <25 mL compared to the experts' largest volume, but the rest of the cases were correctly classified. This results in a classification accuracy of 0.92 (κ = 0.78), and Jacobs and colleagues^14^ observed a kappa coefficient of 0.74 between raters for identification of lesions >25 mL.

For cisterns, icobrain's volumes are in alignment with the experts' reference volumes with an ICC of 0.94. In only 6 of 70 cases, we incorrectly measured cisterns volume >5 mL compared to expert volume and 1 case the other way around. Finally, for the MLS, the average difference between icobrain and the experts' shift measurement was −0.12 mm (median = −0.22), and Jacobs and colleagues^14^ reported the average MLS difference of −0.20 mm between the two experts on their data set. The classification accuracy between icobrain and experts' measurements for the MLS >5 mm was 0.89 (κ = 0.78), and Chilamkurthy and colleagues and Jacobs and colleagues^13,14^ reported the kappa coefficients of 0.60 and 0.82, respectively, among raters. Again, the absolute difference in the shift is moderately high (0.86), but still below the commonly used threshold of 5 mm for MLS. In 3 of 38 cases, we mistakenly measured a shift >5 mm and in 1 case a shift of <5 mm compared to expert measurements. This is similar to a comparison between experts where the maximum disagreement was observed in 5 of 41 cases in the Wang and colleagues study.^41^

The main difficulty in assessing any automated method's performance using only volumes is that it does not incorporate the information regarding the spatial overlap with the segmentations of the expert.^42^ In the extreme case, an automated method can obtain the same volume as the experts with no common voxels. Therefore, in this article, spatial measures such as overlap (Dice), sensitivity, and precision were used for the qualitative assessment of our method against expert segmentation. For example, in the case of acute intracranial lesions and cistern segmentations, a reasonable overlap of >0.70 was attained, which implies that the achieved volumetric performance is acceptable. We observed (results not shown) that the Dice was comparatively low (0.44) when the total cistern volume was <5 mL. This is attributed to the fact that Dice is sensitive to small volumes.^42^ That is why we presented Dice, sensitivity, and precision for cistern volumes >5 mL.

There are some inherent limitations to our study. We occasionally segmented parts of normal blood-containing structures as acute intracranial lesion, which could explain a decrease in precision. However, previous approaches based on intensity thresholding and morphological constraints^34^ had more-severe problems in terms of precision (specificity of 59% for lesion detection, considered at lesion level, not voxelwise). Our method also partly segmented tSAH as lesions, which was not included in the manual segmentation, because it is very time-consuming. However, tSAH also commonly forms extra-axial collections that co-occur with large subdural or epidural hematomas. We missed some hypodense areas in otherwise predominantly hyperdense subdural hematomas, which decreased the sensitivity (undersegmentation) of the method. However, acute bleeding was mostly hyperdense, and the hypodense parts were usually quite small compared to the volume of the entire lesion. For cistern segmentation, our volumetric approach could complement the typical clinical evaluation, which aimed at observing whether each basal cistern was normal, partially compressed, or totally obliterated. The cistern volumes from icobrain can lead to a similar interpretation: First, a cistern is considered compressed when its volume falls under the normal (age-matched) range, which can be obtained from a healthy controls data set; second, a cistern can be considered obliterated if its icobrain volume is negligible.

In the future, we would like to extend the acute intracranial lesions to include edema as well and classify different types of acute intracranial lesions. We also intend to use a convolutional neural network for cistern segmentation and validate our approach on the complete CENTER-TBI data set. These larger numbers will permit more-robust analysis of our method's performance on different scanners and acquisition protocols.

In conclusion, the proposed automatic framework provides reliable quantification of CT features in acute TBI. We believe that, through its robustness and automation, icobrain could bring an added value (possibility to measure acute intracranial lesion and cistern volumes, as well as MLS) for the clinical evaluation of TBI patients (care is advised with its use and interpretation). In addition, this tool can be of great value in large-scale patient studies where lesions are required to be measured.
